# Association of *Blastocystis* ST6 with higher protease activity among symptomatic subjects

**DOI:** 10.1186/s12866-021-02341-9

**Published:** 2021-10-19

**Authors:** Seyed Ahmad Karamati, Hamed Mirjalali, Maryam Niyyati, Abbas Yadegar, Hamid Asadzadeh Aghdaei, Ali Haghighi, Seyyed Javad Seyyed Tabaei

**Affiliations:** 1 Department of Medical Parasitology and Mycology, Faculty of Medicine, Shahid Beheshti University of Medical Sciences, Tehran, Iran; 2 Department of Medical Parasitology and Mycology, Faculty of Medicine, Tehran Medical Sciences, Islamic Azad University, Tehran, Iran; 3 Foodborne and Waterborne Diseases Research Center, Research Institute for Gastroenterology and Liver Diseases, Shahid Beheshti University of Medical Sciences, Tehran, Iran; 4 Basic and Molecular Epidemiology of Gastrointestinal Disorders Research Center, Research Institute for Gastroenterology and Liver Diseases, Shahid Beheshti University of Medical Sciences, Tehran, Iran

**Keywords:** *Blastocystis* sp., Subtypes, Clinical symptoms, Protease activity, Pro-inflammatory biomarker

## Abstract

**Background:**

*Blastocystis* sp. is an anaerobic intestinal protozoan parasite of humans and a wide range of animals worldwide. In the current study the correlation between the cysteine protease activity of clinical samples of *Blastocystis* sp. ST1–3 and 6 with the levels of pro-inflammatory cytokines was evaluated.

**Methods:**

Stool samples were collected from subjects with or without clinical symptoms. All samples were cultivated in DMEM medium. The bacteria were eliminated or reduced in *Blastocystis* sp. positive samples subtypes 1–3 and 6 by a variety of antibiotics and consecutive sub-cultures. To prepare parasite lysate, 1 × 10^5^ *Blastocystis* sp. from each isolate were harvested and lysed using freeze-thaw. Protease activity of each isolate was measured and the gene expression of pro-inflammatory biomarkers in HT-29 cell line sensed by isolates was investigated using quantitative Real-time PCR.

**Results:**

Protease activity assay showed inter- and intra-subtype variations among subtypes regarding the presence of symptoms, while the protease activity of symptomatic isolates was higher than asymptomatic isolates. The highest and lowest levels of protease activity were seen in ST6 and ST2, respectively. However, patterns of the expression of pro-inflammatory biomarkers in HT-29 cell line was different regarding the presence of symptoms and time points. There was no significant correlation between protease activity of different subtypes with the expression levels of pro-inflammatory biomarkers.

**Conclusions:**

Our study indicated a higher protease activity among isolates from symptomatic compared to asymptomatic subjects, suggesting functional role for proteases in clinical symptoms due to *Blastocystis* sp. The lack of correlation between the levels of expression of pro-inflammatory biomarkers with subtypes regarding the presence of clinical symptoms proposes the importance of host-related factors in presentation of clinical symptoms.

## Background

*Blastocystis* sp. is a common anaerobic unicellular protozoan parasite isolated from the gastrointestinal tract of humans and a wide range of animals worldwide [1–3]. The high incidence of this parasite has been reported in tropical, subtropical, and developing countries [1, 2, 4].

*Blastocystis* sp. was described almost a century ago; nevertheless, this organism has remained a controversial protist, which its pathogenic mechanisms are still unclear [5]. *Blastocystis* sp. was firstly considered as a commensal organism, but studies in last decades linked the presence of this protozoan with some intestinal and extra-intestinal clinical manifestations [6–10]. However, the pathogenicity, virulence factors, and other risk factors, which may impact the clinical manifestations related to *Blastocystis* sp., need to be understood.

Although a little is known about the cellular mechanisms of pathogenesis of *Blastocystis* sp., it was demonstrated that inflammation of the intestinal mucosa is an expected result of *Blastocystis* sp. colonization [11, 12]. It was also shown that *Blastocystis* sp. recruits the inflammatory cells in mice colon [13], and manipulates the immune responses and cytokine release in the colonic epithelial cells [14]. Proteases (particularly cysteine protease) seem to play an important role during the pathogenesis of *Blastocystis* sp. [15–18]. Proteases released by some pathogens have been reported to have an ability to induce pro-inflammatory cytokines from host cells [19]. This enzyme complex destroys the human secretory immunoglobulin A (sIgA) and helps the parasite to survive and colonize the large intestine [20]. However, inter- and intra- subtype variations have been linked to the virulence and protease activity of *Blastocystis* sp., which probably reflect the variations throughout the proteases genes among subtypes [21]. In the current study, for the first time protease activity of human-prevalent *Blastocystis* sp. subtypes 1–3, and 6 isolated from symptomatic and asymptomatic subjects was evaluated. As well, the inter-and intra-subtype variations of *Blastocystis* sp. on the pro-inflammatory cytokines were investigated.

## Methods

### Ethical approval

All procedures performed in this study were in accordance with the ethical standards (IR.SBMU.RIGLD.REC.1395.83) released by the Ethical Review Committee of the Research Institute for Gastroenterology and Liver Diseases, Shahid Beheshti University of Medical Sciences, Tehran, Iran. As well, the study was approved by the ethics committee/institutional review board of the Research Institute for Gastroenterology and Liver Diseases, Shahid Beheshti University of Medical Sciences, Tehran, Iran.

Written informed consents were obtained from all subjects and in the case that participants were under the of 16 years, informed consent was obtained from their parents.

### *Blastocystis* isolates

The current study was performed on stool samples were collected from apparently healthy subjects and patients with gastrointestinal disorders without known microbial/non-microbial reasons, from our previous study [22], who referred to the parasitology laboratory located at the Foodborne and Waterborne Diseases Research Center, the Research Institute for Gastroenterology and Liver Diseases, Shahid Beheshti University of Medical Sciences, Tehran, Iran.

### *Blastocystis* cultivation

All stool samples were microscopically investigated for *Blastocystis* sp. and other intestinal parasites. In order to cultivate *Blastocystis* sp., stool samples were cultured in Dulbecco’s Modified Eagle Medium (DMEM) (Gibco, Thermo Fisher Scientific, MA, USA) containing penicillin-streptomycin (Sigma, USA), (1000-unit penicillin and 4 mg/mL streptomycin) supplemented with 10% heat-inactivated fetal bovine serum (FBS, Sigma-Aldrich, USA), and were incubated in an anaerobic condition at 37 °C. The positive samples were sub-cultured every 3–4 days. Samples without any growth for *Blastocystis* sp. after 10 days considered as negative.

### DNA extraction and subtyping

Genomic DNA of culture-positive *Blastocystis* sp. was extracted using total DNA extraction kit (Yekta Tajhiz Azma, Tehran, Iran) and stored at − 20 °C until PCR amplification. The RD5 (5′-ATCTGGTTGATCCTGCCAGT-3′) and BhRDr (5′-GAGCTTTTTAACTGCAACAACG-3′) primers were used for molecular subtyping of *Blastocystis* sp. as mentioned elsewhere [23]. PCR products have been loaded on 1.5% agarose gel and then sequenced using an ABI 3130 sequencer. The obtained sequences were edited by Chromas software and the subtype determination of isolates were done using Basic Local Alignment Search Tool (BLAST; http://blast.ncbi.nlm.nih.gov/) [22].

### *Blastocystis* subtype purification

Purification of *Blastocystis* sp. subtypes and elimination or reducing the number of bacteria were done by a combination of partial purification of parasite by Ficoll gradient (Ficoll-Paque™ PREMIUM) and inoculation of parasites into fresh medium containing active antibiotics. In this study, approximately 40 *Blastocystis* sp. isolates from our previous study [22], subtypes ST1–3, ST6 and ST7 were included that during the serial cultivation ST7 was missed. To identify the antibiotic susceptibility of bacteria in each isolate, 50 μL of culture medium of each isolate was cultivated onto blood agar, followed by disk diffusion assay. Accordingly, based on the antibiotic susceptibility test, a cocktail of antibiotics, specific to each isolate, was used to reduce the number of bacteria. After a six-month consecutive cultivation by specific antibiotics (a mix of antibiotics consisted of 4000 mg/ml of ampicillin, 1000 mg/ml of streptomycin, and 1000 units of penicillin together with amphotericin B (50 mg/ml) to eliminate yeasts or filamentous fungi) for each isolate, culture medium was centrifuged in 250×g at 25 °C for 5 min, supernatant containing bacteria was removed and the remained pellet containing purified *Blastocystis* sp. was washed three times with sterile PBS (pH = 7.5–8) [24].

### Preparation of parasite lysates

Purified parasites without bacteria or with reduced number of bacteria were cultivated in DMEM medium supplemented with 10% heat inactivated FBS, 1000 mg/mL of streptomycin, and 1000 units of penicillin, and then incubated at 37 °C in an Anaerojar (Oxoid, United Kingdom). Two to three-day-old parasites at log phase were washed three times in PBS at 300×*g* for 5 min at 4 °C. The parasites were counted with Neubauer’s improved cell counting chamber (perci color HBG; Germany), and the parasite concentration was adjusted to 1 × 10^5^ parasites/mL. Finally, parasite lysates were prepared using three freeze-thaw cycles in liquid nitrogen and a 37 °C water bath, respectively. In order to remove probable remained bacteria, prepared lysate was filtered using polyethersulfone (PES) filters with 0.22 μm pore size.

### Measurement of the protease activity of the *Blastocystis* sp. subtypes

The protease activity of the *Blastocystis* sp. subtypes in both symptomatic and asymptomatic groups were determined using protease activity assay kit (Abcam, United Kingdom). In brief, 100 μL of parasite lysates was added to 300 μL of the assay buffer and was centrifuged at 400 g for 5 min to remove insoluble materials. Then, 50 μL of the supernatant was added to wells of a 96-well plate (black plate with clear bottoms, SPL). For positive control, 5 μL of the reconstituted positive control solution was transferred into wells, and the final volume was adjusted to 50 μL with assay buffer. A reagent background control containing only 50 μL of assay buffer was also prepared. In order to prepare standard curve, volumes of 0, 2, 4, 6, 8, and 10 μL of FITC standard were added into a series of standard wells, respectively. To generate 0, 0.05, 0.1, 0.15 0.2, and 0.25 nmol/well of the FITC standard, the final volumes were adjusted to 100 μL/well with assay buffer, respectively. The reaction mix containing 2 μL of protease substrate solution plus 48 μL of assay buffer for each well was prepared and added to the wells, except standard wells. Finally, the excitation (ex) and emission (em) of fluorescence of the unquenched FITC generated by proteolytic digestion of the substrates were read at Ex/Em = 485/530 nm. The fluorescence of the unquenched FITC generated by proteolytic digestion of the substrate measured by ΔRFU = R2 – R1 formula.

### Cell culture

The human colon adenocarcinoma cell line (HT-29; ATCC HTB-38), which was kindly provided by the Institute Pasteur of Iran, was cultivated in a 25-cm^2^ culture flask (Cell culture Flask, SPL, Korea) containing 5 mL of high-glucose DMEM medium (DMEM High Glucose, Biosera) supplemented with 5% (v/v) heat-inactivated FBS, 2 mM L-glutamine, penicillin (100 U/ml, Sigma-Aldrich, USA), and streptomycin (100 mg/ml, Sigma, USA) and incubated in 5% CO_2_ and 100% humidity at 37 °C. Subsequently, after 70–80% confluency, the cultured flask was washed three times with sterile PBS (pH = 7) and trypsinized using 0.25% trypsin-EDTA (Gibco, USA). Finally, the cell suspension was diluted in 1:1 ratio with a 0.025% (w/v) trypan blue solution (Gibco, USA) and cell counting was carried out with Neubauer’s improved cell counting chamber.

### Co-incubation of *Blast-*Ag from different subtypes with HT-29 cell line

For co-incubation of *Blast-*Ag and HT29 cell line, 1 × 10^5^ HT-29 cells were seeded in each well of a 12-well plates. The plate was incubated in 5% CO_2_ at 37 °C overnight. After 70–80% confluency, the parasite lysates prepared from 10^5^ of *Blastocystis* sp. subtypes were added to each well and incubated for six, 12, and 24 h.

### RNA extraction, cDNA synthesis, and quantitative real-time PCR

Total RNA extraction was performed using Total RNA Purification Mini kit (YTA, Tehran, Iran). After adjusting the RNA concentration, first-strand cDNA synthesis was performed using cDNA synthesis kit (YTA, Tehran, Iran) according to the manufacture’s instruction. Relative fold differences of the pro-inflammatory biomarkers expression among treated and untreated cells were determined by qreal-time PCR using Rotor-Gene Q (Qiagen, Germany) in a 20 reaction mixture containing 10 μL SYBR Green qPCR master mix 2X (Ampliqon, Denmark), 5 μM of each primer (Table 1), and 1 μL constructed cDNA as template. The amplification conditions were: initial denaturation 95 °C for 10 min, followed by denaturation at 95 °C for 20 s, annealing at 59–61 °C for 30 s, and extension at 72 °C for 20 s. Melt curve analysis was done in order to determine the presence of probable non-specific PCR amplification or primer dimer formation. The expression of the pro-inflammatory biomarkers was calculated by the ∆∆CT. The relative quantification (RQ) of the targets genes, relative to the β-actin mRNA, was calculated using relative expression software tool (REST).

**Table 1:** The sequence of specific primers used in this study

Genes	Sequence (5 ‘to 3’)	Ref
**IFN-ɣ**	TGACCAGAGCATCCAAAAGA CTCTTCGACCTCGAAACAGC	[25]
**IL-12p35**	TTCACCACTCCCAAAACCTGC GAGGCCAGGCAACTCCCATTA	[26]
**IL-8**	TGGCTCTCTTGGCAGCCTTC TGCACCCAGTTTTCCTTGGG	[27]
**IL-6**	CCTTAAAGCTGCGCAGAATG ATTCAATGAGGAGACTTGCC	[28]
**TNF-α**	AGCCCATGTTGTAGCAAACC TGAGGTACAGGCCCTCTGAT	[29]
**TGF-β**	ATGCCCGTATTTATGGAGTT ATTGTCATTTTGGTCTTGCC	[30]
**β-actin**	ATGTGGCCGAGGACTTTGATT AGTGGGGTGGCTTTTAGGATG	[31]

### Statistical analysis

The statistical analysis of the expression of pro-inflammatory biomarker genes, the protease activities of the subtypes, and the correlation of them with each other and the symptoms were performed in IBM SPSS Statistics for Windows, v22 (Chicago, IL, USA) and GraphPad, Prism (version 8.0.2) software. One-way analysis of variance (ANOVA) followed by Tukey test were used to compare the differences between groups. A correlation or simple linear regression analysis used to determine the correlation between protease activity of the *Blastocysis* sp. subtypes and the pro-inflammatory biomarkers expression. One-sample t-test incorporated in GraphPad Prism software (version 8.0.2) was employed to calculate mean ± SD, confidence interval, and statistical correlations. *P*-value 0.05 was considered as statistically significant.

## Results

### *Blastocystis* sp., isolates and axenification

From almost 40 *Blastocystis* sp. positive samples, which were included in purification process, only seven isolates subtypes ST1–3 (from both symptomatic and asymptomatic subjects) and ST6 from symptomatic subject remained alive after serial cultures. In addition, the main clinical symptoms related to *Blastocystis* sp. isolates were gastrointestinal disorders including diarrhea, constipation, nausea, and bloating [22, 24].

### Protease activity of *Blastocystis* subtypes

**Fig. 1:**
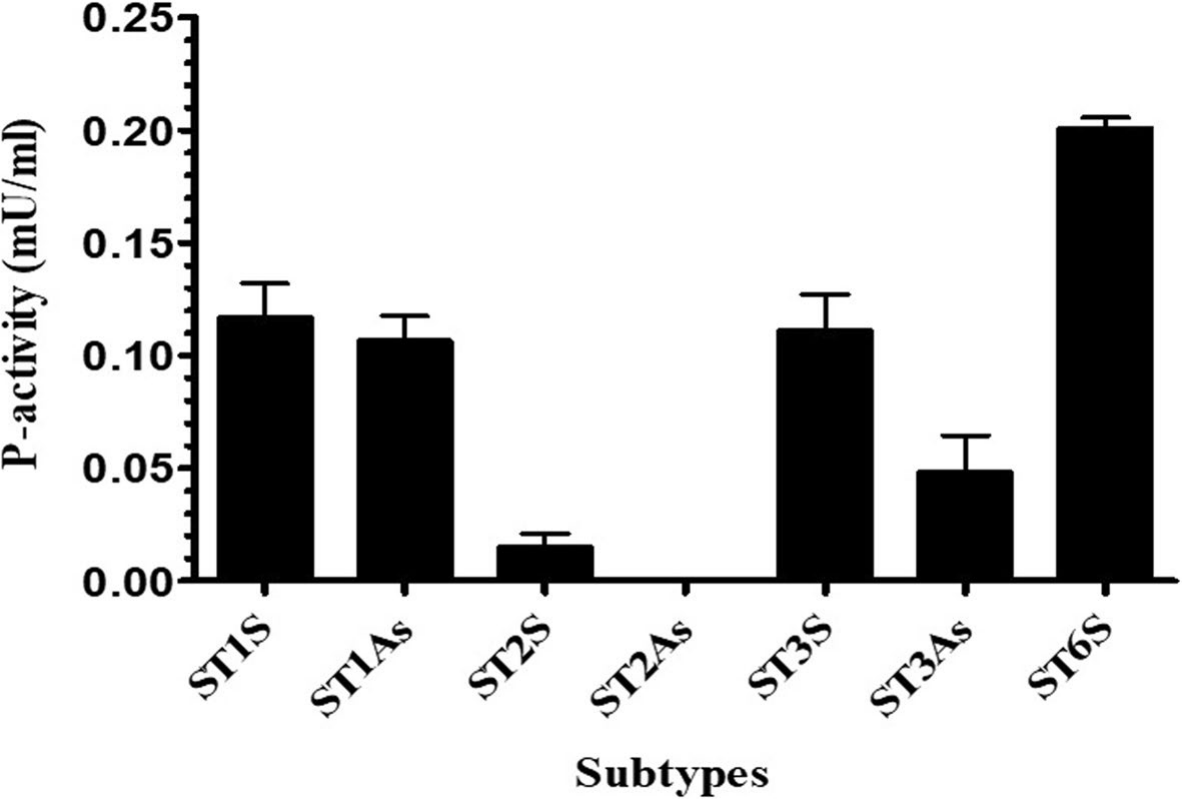
Protease activity of *Blastocystis* sp., isolated from symptomatic and asymptomatic subjects. S: symptomatic; As: asymptomatic

The protease activity of *Blastocystis* sp. isolates from symptomatic isolates was higher than those isolated from asymptomatic subjects. The highest protease activity was seen in ST6 (0.201 mU/mL). Furthermore, ST2 isolated from asymptomatic subject showed no protease activity. Intra-subtype variations in protease activity revealed that there were no significant differences among *Blastocystis* sp. subtypes regarding the presence of symptoms except subtype 3 (*P*-value = 0.014) (Fig. 1).

### The effects of *Blastocystis* sp. subtypes on the expression patterns of pro-inflammatory biomarkers in HT-29 cell line

In this study the expression patterns of selected pro-inflammatory biomarkers including IFN-γ, TNF-α, IL-12, IL-8, IL-6, and TGF-β were evaluated after exposure to *Blast*-Ag in three different time points.

### IFN-γ

**Fig. 2:**
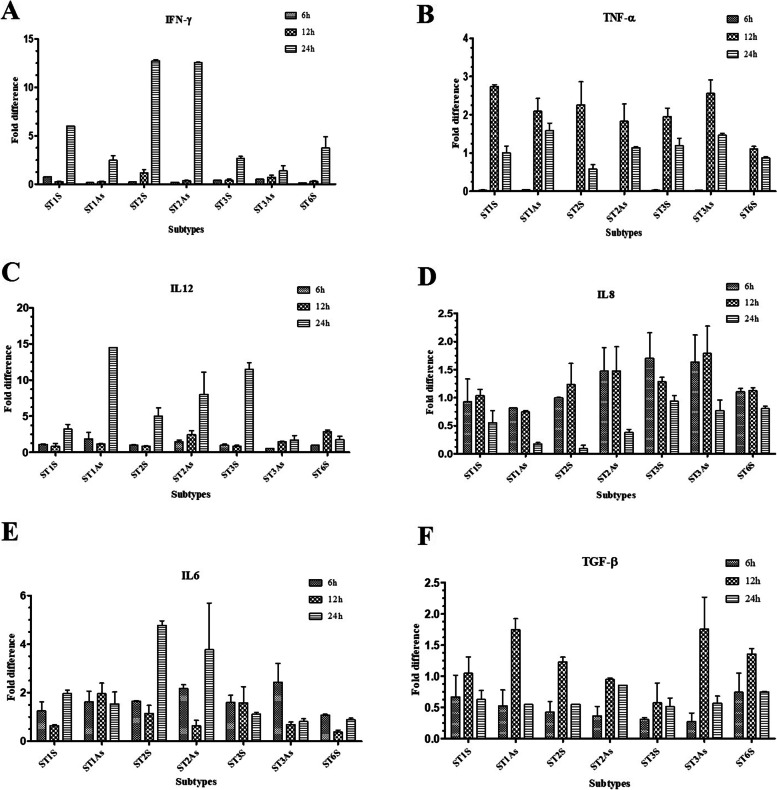
The expression levels of **A)** IFN-γ, **B)** TNF-α, **C)** IL-12, **D)** IL-8, **E)** IL-6, **and F)** TGF-β in HT29 cell line sensing by *Blast-*Ag derived from 1× 10^5^ *Blastocystis* sp., ST1–3 and 6 isolated from symptomatic and asymptomatic subjects

The highest expression level of IFN-γ was seen after 24 h in all isolates with a significant up-regulation in ST2. In addition, intra-subtype variation in the expression of IFN-γ regarding the presence of symptoms showed that almost all subtypes isolated from symptomatic patients presented significant overexpression of IFN-γ after 24 h. A significant overexpression was also seen in ST1 (*P*-value = 0.013) regarding the presence of symptoms, 24 h after exposure to *Blast*-Ag (Fig. 2A, Tables 2 and 3).

**Table 2:** The comparison of the expression levels of pro-inflammatory biomarkers in different time points in symptomatic and asymptomatic isolates

Cytokines	Time	Expression levels							Sig.	***P***-value
		ST1S	ST1As	ST2S	ST2As	ST3S	ST3As	ST6S		
**IFN-γ**	**6 h**	0.775	0.169	0.225	0.198	0.389	0.504	0.131	Yes	0.000
	**12 h**	0.257	0.269	1.1585	0.3665	0.4125	0.7325	0.2745	Yes	0.013
	**24 h**	6.008	2.457	12.7415	12.57	2.684	1.37	3.7405	Yes	0.000
**TNF-α**	**6 h**	0.029	0.038	0.015	0.018	0.03	0.023	0.18	Yes	0.000
	**12 h**	2.732	2.0915	2.26	1.8275	1.9535	2.5635	1.109	Yes	0.046
	**24 h**	1.004	1.588	0.5785	1.139	1.189	1.4645	0.8725	Yes	0.009
**IL-12**	**6 h**	1.102	1.852	0.956	1.43	0.9855	0.539	0.985	No	0.351
	**12 h**	0.825	1.118	0.8245	2.449	0.8735	1.4885	2.8375	Yes	0.002
	**24 h**	3.2	14.451	5.0375	8.035	11.4875	1.714	1.743	Yes	0.000
**IL-8**	**6 h**	0.926	0.816	0.9975	1.4725	1.7025	1.6335	1.104	No	0.395
	**12 h**	1.0375	0.747	1.232	1.4765	1.2855	1.788	1.127	No	0.328
	**24 h**	0.553	0.18	0.0945	0.385	0.9375	0.7685	0.808	Yes	0.003
**IL-6**	**6 h**	1.247	1.6265	1.655	2.1685	1.606	2.438	1.0805	No	0.183
	**12 h**	0.648	1.9585	1.153	0.6335	1.59	0.6925	0.382	No	0.081
	**24 h**	1.9765	1.539	4.7745	3.7695	1.111	0.814	0.8945	Yes	0.027
**TGF-β**	**6 h**	0.6715	0.529	0.427	0.3665	0.3115	0.274	0.7465	No	0.310
	**12 h**	1.049	1.7455	1.227	0.949	0.5745	1.7565	1.3555	No	0.089
	**24 h**	0.629	0.548	0.547	0.856	0.515	0.5725	0.7515	Yes	0.022

**Table 3:** The comparison of the expression levels of pro-inflammatory biomarkers in *Blastocystis* sp., isolates regarding different time points

***Blastocystis*** isolates	Cytokines	Expression levels			Sig.	***P***-value
		6 h	12 h	24 h		
**ST1S**	**IFN-γ**	0.775	0.257	6.008	Yes	0.000
**ST1As**		0.169	0.269	2.457	Yes	0.018
**ST2S**		0.225	1.1585	12.7415	Yes	0.000
**ST2As**		0.198	0.3665	12.57	Yes	0.000
**ST3S**		0.389	0.4125	2.684	Yes	0.002
**ST3As**		0.504	0.7325	1.37	No	0.317
**ST6S**		0.1315	0.2745	3.7405	No	0.053
**ST1S**	**TNF-α**	0.029	2.732	1.004	Yes	0.001
**ST1As**		0.038	2.0915	1.588	Yes	0.015
**ST2S**		0.015	2.26	0.5785	Yes	0.043
**ST2As**		0.018	1.8275	1.139	Yes	0.036
**ST3S**		0.0305	1.9535	1.189	Yes	0.009
**ST3As**		0.023	2.5635	1.4645	Yes	0.007
**ST6S**		0.0185	1.109	0.8725	Yes	0.001
**ST1S**	**IL-12**	1.102	0.825	3.2	No	0.054
**ST1As**		1.852	1.118	14.4515	Yes	0.001
**ST2S**		0.956	0.8245	5.0375	Yes	0.034
**ST2As**		1.43	2.449	8.035	No	0.148
**ST3S**		0.9855	0.8735	11.4875	Yes	0.001
**ST3As**		0.539	1.4885	1.714	No	0.183
**ST6S**		0.958	2.8375	1.743	No	0.052
**ST1S**	**IL-8**	0.926	1.0375	0.553	No	0.509
**ST1As**		0.816	0.747	0.18	Yes	0.000
**ST2S**		0.9975	1.232	0.0945	No	0.069
**ST2As**		1.4725	1.4765	0.385	No	0.176
**ST3S**		1.7025	1.2855	0.9375	No	0.284
**ST3As**		1.6335	1.788	0.7685	No	0.307
**ST6S**		1.104	1.127	0.808	Yes	0.035
**ST1S**	**IL-6**	1.247	0.648	1.9765	No	0.062
**ST1As**		1.6265	1.9585	1.539	No	0.803
**ST2S**		1.655	1.153	4.7745	Yes	0.002
**ST2As**		2.1685	0.6335	3.7695	Yes	0.000
**ST3S**		1.606	1.59	1.111	No	0.675
**ST3As**		2.438	0.6925	0.814	No	0.121
**ST6S**		1.0805	0.382	0.8945	Yes	0.009
**ST1S**	**TGF-β**	0.6715	1.049	0.629	No	0.538
**ST1As**		0.529	1.7455	0.548	Yes	0.026
**ST2S**		0.427	1.227	0.547	Yes	0.025
**ST2As**		0.3665	0.949	0.856	Yes	0.036
**ST3S**		0.3115	0.5745	0.515	No	0.663
**ST3As**		0.274	1.7565	0.5725	No	0.084
**ST6S**		0.7465	1.3555	0.7515	No	0.157

### TNF-α

There was a downregulation of TNF-α 6 h after exposure to *Blast*-Ag. The highest up-regulation of this gene was observed 12 h after incubation among all of subtypes, which down-regulated again 24 h after co-incubation. Apart from ST3, all other subtypes isolated from symptomatic patients have a higher expression compared to isolates from asymptomatic subjects 12 h after incubation (*P*-value = 0.046) (Fig. 2B, Tables 2 and 3).

### Il-12

An up-regulation of IL-12 gene in HT-29 cells was observed for almost all isolates 24 h after exposure to *Blast*-Ag; however, the highest expression of this gene was observed in 24 h in all of *Blastocystis* sp. subtypes excluding ST6S. The highest up-regulation was observed in ST1As (14.45 fold-change). Intra-subtype analysis based on the presence of symptoms revealed that apart from ST3, which IL-12 was significantly upregulated in the isolate from symptomatic patients 12 h after incubation, an overexpression of IL-12 was observed in asymptomatic subjects (*P*-value = 0.002). In addition, the level of IL-12 was significantly upregulated in asymptomatic isolates compared to symptomatic isolates during 24 h after incubation (*P*-value = 0.000) in all samples (Fig. 2C, Tables 2 and 3).

### Il-8

Despite of no considerable expression changes compared to the other cytokines, IL-8 was downregulated in all isolates, particularly in asymptomatic isolates, 24 h after exposure with *Blast*-Ag, which was significantly lower than in 6 h and 12 h after exposure (*P-*value = 0.003) (Fig. 2D, Tables 2 and 3).

### Il-6

The highest expression of IL-6 was seen in both ST2As and ST2S 24 h after exposure to *Blast*-Ag. The expression of IL-6 was significantly increased among symptomatic subjects 24 h after incubation (*P*-value = 0.027) (Fig. 2E, Tables 2 and 3).

### TGF-β

The highest expression of TGF-β was seen 12 h after exposure to *Blast-*Ag in all isolates. In addition, overexpression of TGF-β was observed in ST1 and ST3 isolated from asymptomatic subjects, 12 h after incubation. An increased downregulated of TGF-β was observed in *Blastocystis* sp. isolates from asymptomatic subjects 6 h after incubation with *Blast*-Ag, while it was not statistically significant (*P*-value = 0.31) (Fig. 2F, Tables 2 and 3).

### Correlation between protease activity and expression of pro-inflammatory cytokines

**Fig. 3:**
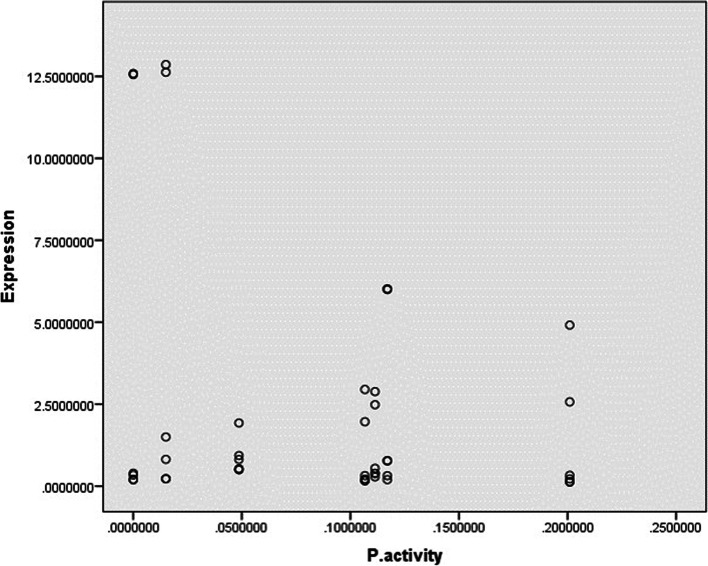
Statistical analysis showed no correlation between protease activity of *Blastocystis* sp., isolated from symptomatic and asymptomatic subjects and the expression levels of pro-inflammatory chemokines (*P*-value = 0.078)

Our study indicated that there was no significant correlation between protease activity of and the expression levels of pro-inflammatory cytokines regarding subtypes and the presence of symptoms (*P*-value = 0.078) (Fig. 3).

## Discussion

The pathogenic role of *Blastocystis* sp. has remained controversially [32–35] and most of studies failed to link the clinical symptoms and presence of *Blastocystis* sp.; therefore, this microorganism is considered as a controversial protist [32, 33, 36]. Many hydrolytic enzymes have been identified and suggested to be important pieces in the pathogenicity of *Blastocystis* sp. [37, 38]. Proteases are considered to play an important role in the pathogenicity of the parasite [35, 39, 40]. Proteases released by *Blastocystis* sp., such as cysteine and serine proteases, can stimulate apoptosis via caspase 3 [41]. These proteases interrupt the barrier functions, increase the permeability of the epithelial cell lines via regulation of the tight junctions, and disrupt actin filaments [18, 42–44]. The proteases can also cleave sIgA, which leads to escape of the parasite from the host immune system, surviving the protist, and colonizing of the gastrointestinal tract [20, 37].

Abdel-Hameed et al. [40] studied protease activities of *Blastocystis* sp. ST3 isolated from symptomatic and asymptomatic humans using gelatin sodium dodecyl sulfate polyacrylamide gel electrophoresis (SDS-PAGE) and found the presence of proteases in 17/18 (94.4%) of symptomatic patients versus 2/8 (25.0%) of asymptomatic people, indicating the importance of proteases in pathogenesis of *Blastocystis* sp. Cysteine proteases secreted by *Blastocystis* sp. are the most important proteases, which are critical in parasite survival and pathogenicity [33, 35]. Therefore, significant variations in cysteine protease activities among *Blastocystis* sp. subtypes may lead to differences in their virulence [42]. In our study, higher protease activity was seen among *Blastocystis* sp. isolated from symptomatic patients compared to those from asymptomatic subjects. This type of differences has been reported from other protists, as well. Reed et al. [45] demonstrated higher cysteine protease activity of pathogenic strains of *Entamoeba histolytica* compared to non-pathogenic strains. Mirza et al. [42] studied the variations in cysteine protease activity among *Blastocystis* sp. isolated from two different hosts, rodent (subtype 4) and avian (subtype 7), and claimed a higher cysteine protease activity among avian isolates. In the line of previous studies, the protease activity of symptomatic isolates of ST1, ST3, and ST6 was significantly higher than that in ST2. Protease activity of the subtype 6 was higher than other *Blastocystis* sp. subtypes in both symptomatic and asymptomatic subjects. This subtype belongs to birds [46], which may induce a high inflammatory process in non-specific hosts [42].

Proteases released by protozoan parasites have an important role in pathogenesis [47] and seem to stimulate secretion of pro-inflammatory cytokines [48]. *Blastocystis* sp. proteases are reported to provoke the immune responses [18, 44]. *Blastocystis* sp. ST1 could arise the pro-inflammatory cytokines including granulocyte–macrophage colony-stimulating factor (GM-CSF) and interleukin 8 (IL-8) [14]. Lim et al. [44] reported that serine proteases released by *Blastocystis* sp. stimulated the production of pro-inflammatory cytokines in murine macrophage cell line. It was proposed that cysteine proteases secreted from *Blastocystis* sp. ST4 could induce the IL-8 production from human colonic epithelial cells (HT84) via a nuclear factor κB (NF-*κB*)-dependent manner [18]. However, a contact-independent cells apoptosis was suggested during the co-incubation of rat intestinal epithelial cells (IEC6) with *Blastocystis* sp. ST4 [41].

In our study, various expression patterns of IFN-γ in HT-29 cell lines were observed among subtypes of *Blastocystis* sp. during three different point times, and a significant up-regulations of IFN-γ was only observed 24 h after incubation. Iguchi et al. [49] observed a high expression of IFN-γ and pro-inflammatory cytokines in the cecal mucosa of rats that were experimentally infected with *Blastocystis* sp. strain RN94–9. In addition, Chan et al. [50] reported a significant up-regulation of IFN-γ cytokine in HCT-116 cell line followed by exposure to *Blast*-Ag derived from symptomatic patients. These studies support our results that showed higher expression of IFN-γ in subtypes 1, 3, and 6 isolated from symptomatic subjects.

Up-regulation of TNF-α gene in HT-29 cell lines by *Blastocystis* sp. subtypes implied that both Th1 and Th2 responses have important roles in the immunity against *Blastocystis* sp. These results are in accordance with Chan et al. [50] who suggested activation of the cellular and humoral immune responses against *Blastocystis* sp. Up-regulation of TNF-α in this study suggested that released proteases by protozoan parasites such as *Blastocystis* sp. could provoke the activation of T cells, monocytes/macrophages, and natural killer (NK) cells, which have important roles in the production of pro-inflammatory cytokines such as TNF-α, IL-6 and IL-1, particularly during the acute phase of infection [51–53].

In our study, up-regulation of IL-12 gene was seen in HT-29 cells upon the exposure to *Blastocystis* sp.; however, the highest expression of this gene was observed after 24 h incubation. In our study up-regulation of IL-12 gene in HT-29 cell lines in contact with subtype 1 isolated from asymptomatic subjects (ST1As) was significantly higher than other isolates. The reason for this observation is not clear. Interestingly, the expression patterns of IL-12 gene in HT-29 cell lines in three point times were similar to IFN-γ. The significant up-regulation of IL-12 and IFN-γ were seen in 24 h after incubation, which implies the important role of the cellular immune system against *Blastocystis* sp.

Type 1 cytokines such as IL-12 have a critical role in cell-mediated immune responses against variety of pathogens, in particular intracellular pathogens [54]. IFN-γ provides a strong stimulation signal for switching the monocytes to activated macrophages, which leads to production and release of IL-12 from macrophages. In contrast, IL-12 stimulates the differentiation of naive T cells into Th1 cells, which encourages the production of inflammatory cytokines such as IFN-γ [55]. In response to antigenic stimulation, IL-12 is naturally produced by the immune system cells including macrophage, dendritic cells, neutrophils, and human B-lymphoblastoid cells and plays a crucial role in the generation of Th1 immune responses [54, 56, 57].

As a result, *Blastocystis* sp., subtypes isolated from both symptomatic and asymptomatic subjects can stimulate the expression of IL-8 gene in HT-29 cell lines. This result is in the line of Lim et al. [44], and implies the potential role of *Blastocystis* sp. in provoking the inflammatory factors during early stages of infection. Long et al. [14] proposed the ability of *Blastocystis* sp. in modulation of IL-8 response in intestinal epithelial cells. In addition, Puthia et al. [18] demonstrated that cysteine proteases secreted by *B. ratti* WR1, a zoonotic isolate, can modulate IL-8 gene expression in human colonic epithelial cells. IL-8 is a CXC chemokine that has an ability in attraction of polymorphonuclear leukocytes into inflammation site, which activates the monocytes and has a role in pathogenesis of inflammatory diseases [58, 59]. However, in the line of previous published study [60] our results suggest that intestinal inflammation induced by *Blastocystis* sp. is mediated by recruitment of inflammatory cells such as IL-8, particularly at the first phase of infection. The recruitment of IL-8 into specific site of infection is necessary to trigger the inflammatory processes, which influxes the inflammatory cells into the intestinal mucosa, leading to gastrointestinal disturbances and tissue damage [61].

We observed a diverse patterns of IL-6 expression in HT-29 cell lines. IL-6 has a dual functional role in Th1/Th2 immune system differentiation [62]. Our results are in accordance with Chan et al. [50] who reported a significant expression of IFN-γ and TNF-α, IL-6, IL-8, and TGF-β in HCT116 upon the co-incubation with *Blasto*-Ag isolated from symptomatic subjects. Lim et al. [44] showed an increased average expression of inflammatory cytokines such as IL-1β, IL-6, and TNF-α in macrophages in response to *Blastocystis* sp. ST7. These studies support our results that *Blastocystis* sp. without considering the subtypes, stimulates the expression levels of IL-6, particularly at 6 h and 24 h after co-incubation.

The high expression of TGF-β gene were seen during 12 h after co-incubation compared to other time points in all samples. This result is in accordance with Chan et al. [50] who reported a significant expression of TGF-β in HCT116, while the up-regulation of TGF-β gene was seen in subtype 1 and subtype 3 isolated from asymptomatic subjects, 12 h after incubation.

## Conclusions

In the current study, high protease activity of *Blastocystis* sp. subtypes isolated from symptomatic subjects compared to asymptomatic subjects indicates a potential role for proteases in pathogenesis of *Blastocystis* sp. In addition, high protease activity of subtypes 6 compared to other subtypes indicates that subtypes that are not human-prevalent and are usually reported from animals may cause more severe symptoms in humans due to their higher protease activity than human-adapted subtypes. As a result, the lack of significant relationship between protease activity of *Blastocystis* sp. subtypes and the expression levels of pro-inflammatory biomarkers suggests that either *Blastocystis* sp.- or host-related factors besides proteases participate in the stimulation of pro-inflammatory biomarkers.

## Data Availability

Generated data including figures and tables were not submitted elsewhere and are included in the article.

## Abbreviations

DMEM: Dulbecco’s Modified Eagle Medium
FBS: Fetal Bovine Serum
FITC: Fluorescein isothiocyanate
*Blast-Ag*: *Blastocystis* antigen
cDNA: complementary DNA
β-actin: Beta actin
mRNA: messenger RNA
ST: Subtype
IFN: Interferon
IL: Interleukin
TGF: Tumor growth factor
